# Severity of vehicle-to-vehicle accidents in the UAE: An exploratory analysis using machine learning algorithms

**DOI:** 10.1016/j.heliyon.2023.e20694

**Published:** 2023-10-05

**Authors:** Praveen Maghelal, Abdulrahim Haroun Ali, Elie Azar, Raja Jayaraman, Kinda Khalaf

**Affiliations:** a Faculty of Resilience, Rabdan Academy, Abu Dhabi, United Arab Emirates; b Industrial and Systems Engineering, Khalifa University, Abu Dhabi, United Arab Emirates; c Civil and Environmental Engineering, Carleton University, Ottawa, ON, Canada; d Biomedical Engineering and Health Engineering Innovation Center, Khalifa University, Abu Dhabi, United Arab Emirates

**Keywords:** Crashes, Severity, Machine learning, Random forest, United Arab Emirates

## Abstract

The World Health Organization (WHO) identifies road traffic injuries as a global health problem. The Eastern-Mediterranean region is particularly suffering from low traffic safety levels, recording the third highest death per capita ratio in the world. It is critical to evaluate and understand the causes of crashes and their severity levels as a first step to devising policies that aim to reduce these causes. Previous studies examining the frequency or severity of crashes present important limitations that motivate the need for the current work. While these studies have investigated the relation of contributing factors to severity of crashes, not until recently the importance of these factors are bring investigated. Even then, less research have explored various Machine Learning models and none in the middle-eastern region. This is critical because the WHO report concludes that the chances of dying in a traffic crash in this region are second only to Africa per 100000 population.

This is a first study analyzing the severity of vehicle-to-vehicle crashes among drivers in the United Arab Emirates. Traffic Crash Data was obtained from the Abu Dhabi Police, which consisted of 11,400 observations during the period 2014–2017. Machine learning algorithms, including gradient boosting (GB), support vector machines (SVM), and random forest (RF), were trained and tested to predict crash severity and extract (using feature analysis) its determinants. The models were evaluated using two performance metrics: prediction accuracy and F1-scores.

The RF model outperformed both GB and SVM, with the confusion matrix of RF reporting a better prediction for all four crash severity classes. The feature importance analysis indicates that the age of car, age of the injured, and the age of the initiator have the highest effect on severity, which is an important finding as the listed factors were rarely considered in previous studies. Vehicle and road characteristics such as vehicle class, crash type, and lighting are slightly associated with the severity. Consistent with other studies, gender was the least essential predictor of severity. Recommendations are finally provided to the Abu Dhabi Department of Municipalities and Transport (AD-DMT) authority to guide the development of road safety policies and countermeasures to mitigate the occurrence and severity of crashes.

## 1 Introduction

The World Health Organization (WHO) identifies road traffic injuries as a global health problem. Urgent action is needed to deal with this devastating health and socioeconomic challenge, which unless addressed head-on, traffic crashes are predicted to become the seventh leading cause of death by 2030, especially among those aged between 15 and 29 years of age [1]. In response, the Sustainable Development Agenda 2030 proposes reducing the global deaths and injuries from road traffic crashes to half by 2030. This is an ambitious target considering that about 1.25 million individuals are killed every year due to traffic crashes. At the same time, 20–50 million suffer non-fatal injuries, costing most countries about three percent of their gross domestic product [2].

Locally, in the United Arab Emirates (UAE), about 57% of crashes and road injuries occur with the driver/passenger of 4-wheeled vehicles [3]. The WHO report concludes that the chances of dying in a traffic crash in this region are second only to Africa per 100000 population. In the UAE, there are 5.5 deaths in traffic crashes per 100,000 population, half of which involves youth aged between 18 and 30 years old. Also, witnessing an increasing trend 725 lives were lost in year 2016 in comparison with 675 during the year 2015 [4]. The Health Authority of Abu Dhabi [5] reports that death due to crash injuries in Abu Dhabi has increased by 10 % from 2014 to 2015, with crash injuries being the second leading cause of death (21.3 %) and healthy life years lost. Fatal injuries are caused primarily by road traffic crashes (47.2 %), followed by poisoning (11.3 %) and falling objects (7.3 %).

Studies investigating road crashes in the middle-east and more-so in the UAE are limited at best [6]. Studies thus-far have either surveyed the residents of Abu Dhabi to assess traffic accidents [7], or have used descriptive approaches based on crash data obtained from the Directorate of Traffic [8]. Studies that investigate the relation of various driver and crash characteristics use the traditional statistical models (Logistic Regression, Binomial Regression) to analyze the road crashes in the UAE [9]. A study by Taamneh et al. [10] employed data-mining techniques such as Decision Tree, Rule induction, Naïve Bayes, and Multilayer Perceptron methods to assess traffic crashes in the UAE. However, they reported no major difference across the models. While these studies analyzed the traffic crashes, none investigated the severity of crashes in the UAE. Also, this study is one of the first that analyzes and compares the efficiency of various Machine Learning (ML) algorithms to understand the important factors related to severity of crashes in the UAE.

Previous research has classified the severity of crashes either as a binary measure (fatality or injury) [11], tertiary measures [12] or categorized into 4 levels such as fatality, serious injury, moderate injury, and mild injury [13]. Irrespective of the categorization, most studies use measures broadly grouped into environmental, personal, and vehicular characteristics when examining the severity of crashes [14]. Environmental factors include weather conditions, visibility, illumination, time of the day, road characteristics, or road geometry [15,16]. The road characteristic is considered a critical environmental factor (e.g., if the road is straight, curved, inclined, on a bridge, or under a tunnel). Another vital road characteristic is the number of lanes and whether the road has multiple or one-way lanes. According to Wang et al. [17],: “increased number of lanes would increase risk of crashes; and improved road infrastructure can reduce crashes”.

Based on the literature, Motor Vehicle Collisions (MVCs) occur due to several personal factors. These factors include erroneous judgments/actions by road users, road conditions, weather conditions, and vehicle conditions [18]. However, as observed from the data from several countries, driver errors are the cause of high percent of road crashes [19,20]. Among the driver characteristics, age plays a significant role, as evident in various studies. Eustace and Wei [21] determine that younger drivers (16 through 19 years of age) and the elderly (75 years and older) were responsible for a disproportionate number of fatal crashes. Also, studies across the world report that wearing seatbelt is crucial to reduce fatal crashes [22]. For instance, a study in Russia found that seatbelts prevent about 50 % of fatal motor vehicle crashes [23]. According to Road Safety studies [24], 40–65 % of death and serious injuries can be reduced by wearing seatbelts in the front seats, and up to 80 % for children if restrained properly in their seats.

Vehicle characteristics include various factors such as vehicle size and type (i.e., passenger car, SUV, truck, van, heavy or light vehicle, and motorcycle). A study on how the type and the size of vehicles affect MVCs found that large vans had the lowest occupant fatality rate at 9.34 per 100,000 vehicles, while compact cars had the highest fatality rate of 17.76 fatalities per 100,000 registered vehicles [25]. In the UAE, the most common type of vehicle is the SUV (or 4*4 cars), which increases the amount of tissue damage associated with injury due to the increased momentum. Additionally, overloaded heavy trucks have been reported to cause risk for other road users in the UAE [26].

Predictive models of crash severity have been extensively investigated in the literature. The most common models used to study crash severity include logit and probit models investigating different associated factors such as environmental, vehicle factors, driver profile, and road conditions [27,28]. When the outcomes are discrete variables, it is recommended to use binary variables for severity; hence, the most used models are the ordered probit model (OPM), the binary logit model (BLM), and the ordered logit model (OLM). On the other hand, if the severity levels are categorized as fatality, severe, moderate, and mild, the OPM and OLM models are commonly used. Moreover, many researchers studied the effects of the number of crashes and variables on the selected model. However, no significant statistical differences between logit, probit and other models were observed [27]. Furthermore, crash severity has been studied using nominal or ordinal models; common nominal models are multinomial logit models, nested logit models and mixed logit models, while ordinal models include ordered logit models, ordered probit models and ordered mixed logit models [29]. While the traditional regression approach investigates the linear and non-linear relationship between the independent and outcome variables, recently, the ML-based feature analysis is extensively used to understand the level of importance of the factors impacting the outcome. However, studies comparing the traditional models with ML models report contrary result. While some studies report ML algorithms of Random Forest reporting higher accuracy [30], other studies report that the traditional models report the best performance [31]. While our current study doesn't involve a comparison with traditional models, it focuses on evaluating various ML models to identify models that demonstrates superior performance. This identified model can serve as a valuable tool for future investigations into crash severity, applicable not only in Middle Eastern nations but across diverse regions globally The proposed methods can also be used to analyzes pedestrian-vehicular accidents, public and private auto crashes and include measures of geographical, social and political setting of the study area. Also, fiscal impact of these crashes can be analyzed using the measures of vehicle and individual characteristics with the insurance claims as outcome variables for future studies.

This study is an exploratory analysis of various environmental, individual, vehicular, and behavioral factors with the severity of Vehicle-to-Vehicle (VHV) crashes in Abu Dhabi (UAE). The study analyzes the severity of the crashes using machine learning-based algorithms such as Random Forest (RF), Gradient Booster (GB), and Support Vector Machines (SVM). While most studies which predict the severity of crashes have used parametric approaches, such as ordered logit or probit models of Multinomial Logit Models, the underlying assumptions and predefined associations can yield misleading results [13]. ML algorithms, on the other hand, are effective in capturing the non-linear effects of both the continuous and discrete variables and achieve higher predictive accuracy [13]. Moreover, given the data restrictions, understanding such investigations from the UAE is limited, if not unavailable. This study thus provides insights into a less explored region in the literature, with implications that can be contextualized within the Gulf Cooperative Countries (GCC), as well as the MENA (Middle-East and North Africa) region that share similar social, geographical, and cultural contexts to those of the UAE. Such studies are relevant not only to the UAE, but also to other GCC countries due to their similarities in road design, vehicle fleet, and driving culture [6].

The following section describe our data collection and the data modeling techniques. The variable measurement and its imputation is discussed followed by the description of the general characteristics of each of the three models used for this study. We then report our results based on the importance of the three models using the three group of variables: Individual characteristics, road and crash characteristics and the vehicular characteristics. Predictive performance of the three ML models are compared using model characteristics and outcome. Next, we interpret our findings and discuss the implications for enabling better road safety in the UAE and the GCC region. Also, application and benefits of using the proposed methods for future research is discussed.

## 2 Methods

**Fig. 1:**
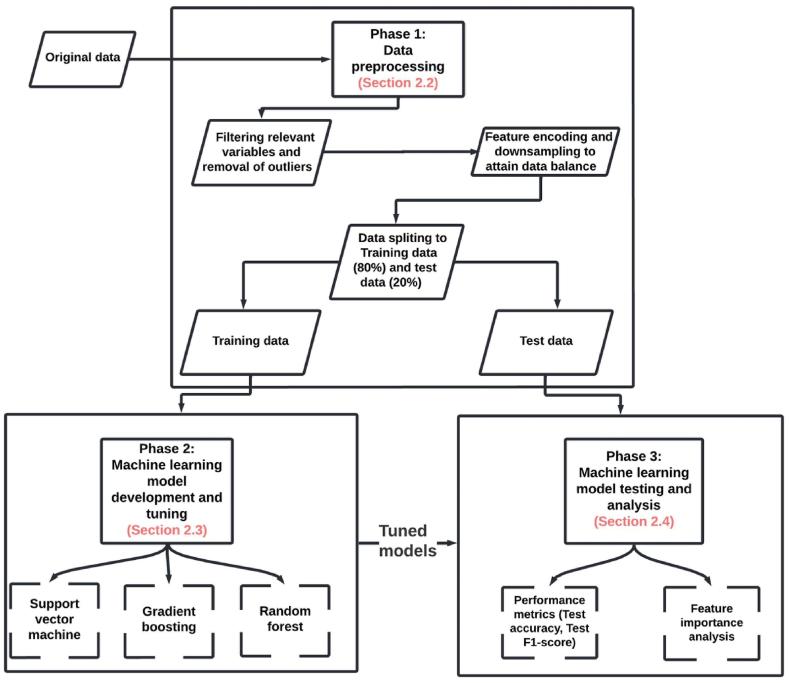
Methodology framework.

The methodology is divided into four phases, as depicted in Fig. 1 and detailed in the following sub-sections. Section 2.1 presents the dataset description while Section 2.2 discusses data preprocessing steps. Section 2.3 describes the model development and tuning phase, while Section 2.4 details the ML models’ testing and analysis of their predictive performance.

### 2.1 Dataset

Data for this study was provided by the Abu Dhabi Police (ADP), excluding the personal identifier information upon request. The data contains a total of 11334 individuals injured in various accidents recorded in the Emirate of Abu Dhabi from 2014 to 2017. The number of individuals per accident ranged from 1 (single vehicle accident) to 23 (multi-vehicle collision) with a total of 8901 injured individuals with varying degree of severity of crashes. The record also include information related to the socio-demographic information of injured person, crash location and description, environmental factors at the time of the crash, and traffic factors (e.g., if the road traffic is moving or congested). Based on the crash data available, the frequency of crash severity in Abu Dhabi from 2014 to 2017 is 3870 for mild injuries, 5640 for moderate injuries, 831 for severe injuries, and 993 for fatal crashes.

### 2.2 Data preprocessing

The data preprocessing was conducted in two steps to align the data to meet the research objectives of this study. Firstly, this study mainly focuses on vehicle collisions; therefore, crash entries unrelated to vehicle crashes were removed. Examples of such entries include data related to motorcycle or bicycle crashes. Secondly, values noted as outliers led to the deletion of the whole row. For example, both *initiator age* and *injured age* had values over 500 years, deemed unrealistic and deleted. The final datasets following the steps above consisted of 7853 samples (rows) with 15 variables, including 14 independent variables and one dependent variable (“*Degree of injury*”), as shown in Table 1.

**Table 1:** Independent and dependent variables.

	Variable name	Type	Feature engineering	Imputation method
Independent variables	*Road and Crash Characteristics*			
	Crash reason	Categorical	One-hot encoding	Probabilistic
	Road type	Categorical	One-hot encoding	Probabilistic
	Lighting	Categorical	One-hot encoding	Probabilistic
	Intersection	Categorical	One-hot encoding	Probabilistic
	Crash type	Categorical	One-hot encoding	Probabilistic
	Road speed	Continuous	None	Average
	Using the belt	Categorical	One-hot encoding	Probabilistic
	*Individual Characteristics*			
	Initiator age	Continuous	None	Average
	Injured age	Continuous	None	Average
	Injured gender	Categorical	One-hot encoding	Probabilistic
	Initiator gender	Categorical	One-hot encoding	Probabilistic
	*Vehicular Characteristics*			
	Vehicle class	Categorical	One-hot encoding	Probabilistic
	Standard manufacturing year	Continuous	None	Average
	Car brand	Categorical	One-hot encoding	Probabilistic
Dependent variable	Degree of injury	Categorical	Ordinal encoding	None

**Fig. 2A:**
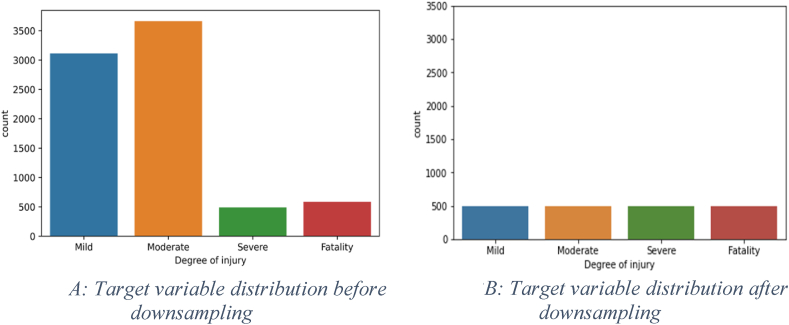
Target variable distribution before downsampling Fig. 2B: Target variable distribution after downsampling.

Additionally, categories that are less presented (less than 10 %) in a variable were merged and categorized as “Others”. For example, “*road type”* had nine categories, many of which had less than 10 % representation. Hence, only the top 3 (over 10 % representation) were kept and the rest were merged into an “Others” category. The main goal of this process is to combine the effect of less-represented types in the model learning process to avoid unnecessarily complex models to train and test. All categorical independent variables are encoded using one-hot encoding, while the dependent variable is encoded using ordinal encoding. One-hot encoding refers to generating a separate dummy binary variable for each category within the categorical variable. For each particular category, only the dummy variable representing that category is assigned a value ‘1’ while the remaining dummy variables are assigned value ‘0’. On the contrary, ordinal variables contains categories that indicate an increase or decrease of intensity of values. Hence, ordinal encoding assigns decimal numbers to each category in an order to capture the ordinal measure. The subsequent step involves examining the data for possible imbalances, an undesirable condition in machine learning that can impact the interpretation of model's performance [32]. As shown in Fig. 2A, the data indicated a considerable imbalance in its target variable. Hence, a downsampling approach was implemented to achieve a more balanced distribution. Downsampling is a common technique employed to handle imbalanced datasets by randomly choosing a subset of samples from the majority classes to provide balance with the minority class [33]. Following this approach, all 490 samples of the *Severe* category were kept, which is the least represented category (i.e., minority class). Then, 490 samples were randomly selected from the *Fatality, Mild*, and *Moderate* types, which are more represented (i.e., majority classes). The resulting Downsampled dataset contained 1960 samples (rows), as illustrated in Fig. 2B. The data is then divided into a *training dataset*, which refers to the data subset that is used for training the model, and a *testing dataset*, which refers to the data subset used to test the model. An 80/20 training/testing split was adopted, which is commonly used in ML applications [34]. The split is performed in a stratified manner in which an equal number of categories is observed in both the training and testing sets.

Any missing values for the stated variables are imputed using one of two approaches, depending on the variable type. “Categorical” variables are assigned following a probabilistic function, which maintains the original distribution of the data. “Continuous” variables are imputed using the average of the existing responses. It is worth mentioning that the imputation was done after splitting the dataset into training and testing sets to avoid “data leakage”. Data leakage refers to information from the testing dataset influencing the training dataset [35]. For instance, if data imputation was performed using average values obtained from the entire dataset, information from the (soon-to-be) testing dataset would influence the imputed values in the (soon-to-be) training dataset.

### 2.3 Machine learning model development and tuning

Three popular machine learning algorithms are selected: Random Forest ([RF),], Gradient Boosting ([GB),], and Support Vector Machines ([SVM),], as described in Table 2. The chosen techniques are popular and well documented in the literature, with proven predictive abilities for classification problems [36].

**Table 2:** Machine learning algorithms hyperparameter ranges.

Algorithms	Hyperparameter	Hyperparameter range, adapted from [[37], [38], [39]]
Random Forest [RF]	Number of trees in the forest.	11 values in the range of 100–1200.
	Maximum depth for each tree.	10 values in the range of 3–15.
	Minimum samples required for each leaf.	10 values in the range of 1–10.
Gradient Boosting [GB]	Number of trees in the forest.	11 values in the range of 100–1200.
	Maximum depth for each tree.	10 values in the range of 3–15.
	Learning rate [contribution of each tree to the final output].	9 values in the range of 0.01–0.5.
	Minimum samples required for a leaf to be allowed to split further.	8 values in the range of 2–15.
Support Vector Machine [SVM]	Regularization term.	l1, l2, elescticnet [both values].
	Alpha value [multiplied with regularization term].	17 values in the range of 1e-7 to 0.5.
	L1 ratio [considered when “elasticnet” regularization is used].	Ranges from 1e-10 to 1.
	Learning rate value.	Ranges from 0.001 to 0.5.
	Learning rate schedule.	Optimal, constant, invscaling, adaptive.
	Intercept estimation.	True, False.

The experiment was conducted using a low code python-based library known as PyCaret [31] in a Jupyter Notebook environment executed using Dell Precision T5810 2.8 GHz. PyCaret is commonly used to develop ML algorithm in various studies in literature [[40], [41], [42], [43]]. All three models were tuned using accuracy metric, which is a default metric in the PyCaret tool when tuning classification type of ML problems.

Random forest (RF) is an ensemble technique that relies on the bagging approach. The bagging approach combines both the bootstrapping and aggregate techniques. Bootstrapping consists of randomly sampling the data from the original data in building each tree. The aggregate technique performs predictions using the majority class voted by all the trees [44]. Typically, the generated classifiers (in this case decision trees) need to be as less correlated. In RF models, this is typically attained by the bootstrapping (intoduces variability along the data samples) and random subspaces (which introduces variability along features). The correlation between tress impacts the variance of the ensemble as shown in equation below [1] which is a function of correlation between trees ρ, variance of each tree σ^2^, and number of trees in the forest B. Although the number of trees is inversely proportional to the ensemble variance, caution on the number of trees should be taken to avoid highly correlated trees as the number of trees increases and an obvious increase in computational cost.

[1] $$variance\;of\;the\;ensemble=\rho \;\sigma 2+\frac{1-\rho }{B}\sigma 2$$

**Fig. 3:**
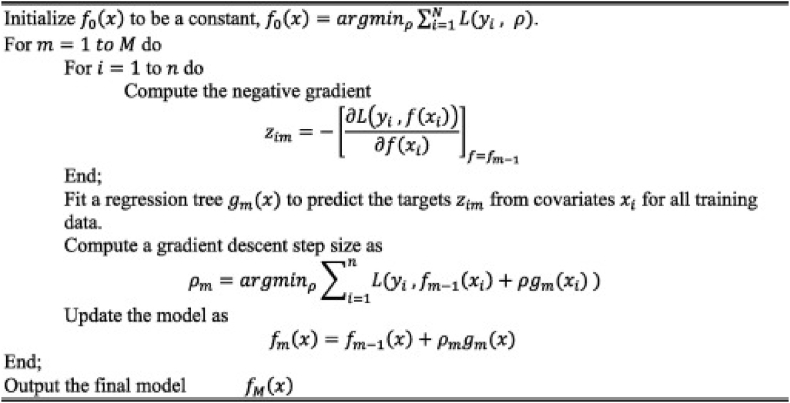
Gradient boosting model pseudocode (Adapted from Ref. [45]).

Gradient boosting (GB) is an ensemble technique that relies on boosting approach whereby several weak learners are iteratively generated and combined to form a single strong learner [45]. Weak learners which are decision trees are combined in an additive weighted manner to make a final prediction. Pseudo-residuals are generated by differential a loss function with respect to the predicted value as shown in Fig. 3 below. Unlike RF which generates tree to predict the target variable, GB instead generates tree to fit the residuals. By gradient decrease of residuals, each generated tree is an improved version of the previous tree.

**Fig. 4:**
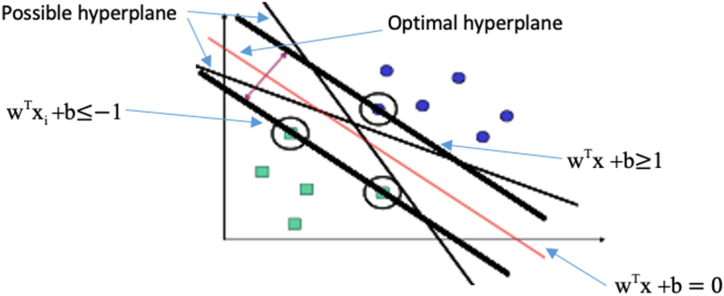
SVM algorithms (Adapted and modified from Ref. [46]).

Support vector machine (SVM) is an ML technique that generates a hyperplane that separates two classes as shown in Fig. 4. The optimal hyperplane is the one that maximizes the margin between the two classes and the points falling on the margin are known as support vectors. While optimizing the weights of hyperplane that maximizes the margin, SVM is constrained to keep each data sample within its appropriate boundaries (wTx + b ≥ 1 for positive samples and wTxi + b ≤ −1 for negative samples). Data samples that falls within the boundaries are known as support vectors.

While hard margin SVM refers to the strict separation of two classes, soft margin allows some misclassification but assign penalty for each missclassifed sample. Hence, the objective function minimizes the penalty via a losses function and maximizes the margin. Given, some of the real world data is hardly linearly separable, soft margin SVM is greatly applicable. In the case of non-linearly separable data, the data is transformed to higher dimensions using the “kernel technique” that eventually becomes linearly separable [46].

The ML algorithm development phase requires setting up the initial experiment parameters that will impact the model performance. Each ML algorithm has its own set of hyperparameters that must be tuned to maximize its predictive accuracy and generalizability. The selected hyperparameters for RF, GB, and SVM with their defined ranges are presented in Table 2. Each model is tuned using a random search using 1000 iterations. Random search does not test all hyperparameter combinations. Instead, it randomly selects a defined number of hyperparameter combinations, rendering it less expensive than a full grid search while still producing competitive results [37]. To avoid overfitting, a cross-validation approach was incorporated with 10 folds.

### 2.4 ML model testing and analysis

This section highlights how the tuned models are evaluated and analyzed. It starts by testing the tuned model with previously unseen data ([i.e., testing dataset).]. The performance of each model is evaluated using the accuracy and F1-scores, referred to as the *test accuracy* and *test F1-score,* respectively.

Accuracy refers to the ratio of correctly classified samples to the total number of samples, usually presented in percentages. As seen in Equation (2), accuracy is derived using four main metrics: [i] true positive [TP] [correctly classified positive samples], [ii] true negative [TN] [correctly classified negative samples], [iii] false positive [FP] [incorrectly classified negative samples], and [iv] false negative [FN] [incorrectly classified positive samples].

[2] $$Accuracy=\frac{TP+TN}{TP+TN+FP+FN}$$

The F1-score is the harmonic mean of two values, precision and recall. Precision measures the model's ability to predict TP values from all predicted positive values [TP and FP]. Recall measures the model's ability to predict TP values from the actual positive values [TP and FN]. Formulas used in calculating precision, recall, and f1-score are shown in Equations [3], [4], [5], respectively.

[3] $$F1\_score=\frac{2\times Precision\times Recall}{Precision+Recall}$$

[4] $$Precision=\frac{TP}{TP+FP}$$

[5] $$Recall=\frac{TP}{TP+FN}$$

When tuning the models, a cross-validation technique was used; hence, the reported metrics are denoted as *cross-validation accuracy* and *cross-validation F1-score*, respectively.

The best-performing model typically has the highest test accuracy and F1 score. Both metrics are scaled between 0 and 1 whereby 0 indicates worst performance and 1 indicates a perfect score. Once the best-performing model is identified, further analysis was conducted to determine its main determinants (i.e., drivers). Feature importance is applied, a technique used to assign a score for each feature based on its contribution to model predictions. Several approaches are available to compute feature importance based on the type of ML algorithms used. Tree-based models, such as RF and GB, often use the *Gini Importance* metric [47], while linear SVM models typically use the *coefficients* of the features used as predictors in the weighted linear equations of the model [48].

## 3 Results and discussion

### 3.1 Descriptive analysis

**Fig. 5:**
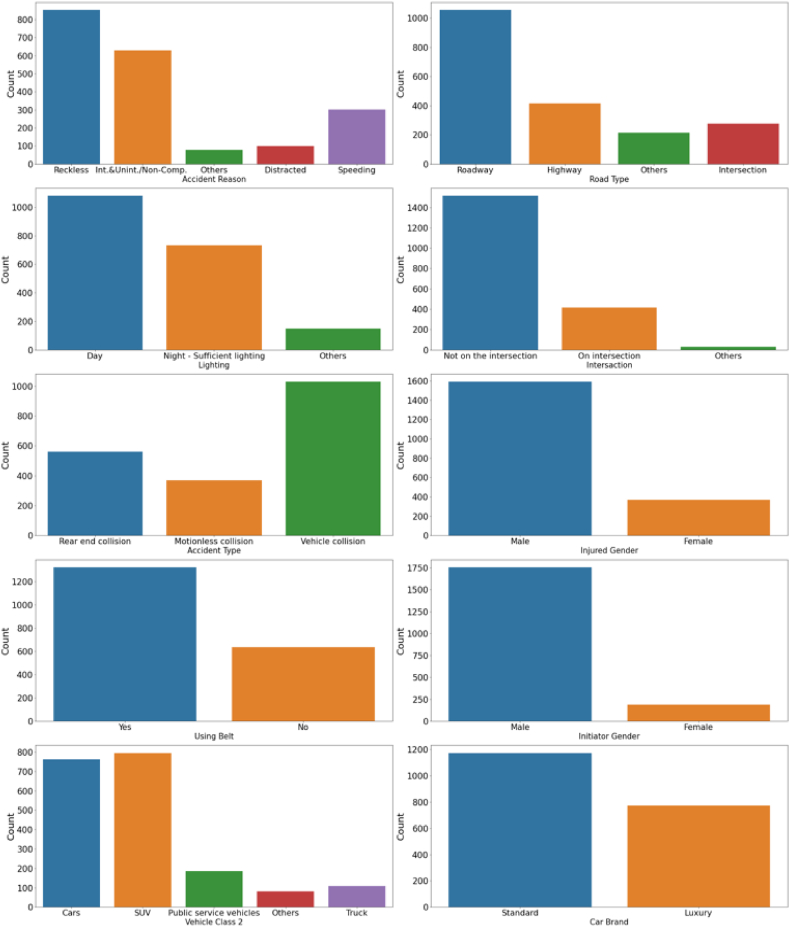
Histograms of categorical variables.

**Fig. 6:**
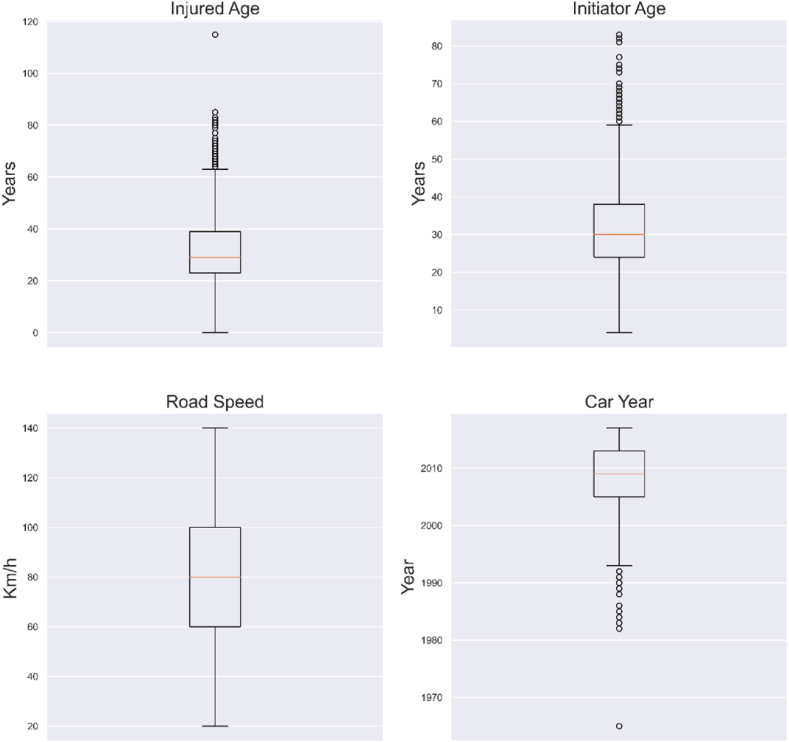
Boxplots of continuous variables.

The categorical variables' distribution is illustrated in Fig. 5, while the statistics for the continuous variables are shown in Table 3 and Fig. 6. The data is representative of the region (UAE, GCC and the MENA context) and reflects the driving behavior typical in the country. The three main context-relevant data are the reasons for crashes, the gender of the initiator and the injured, and the vehicle class. Fig. 5 indicates that the top two reasons for crashes are reckless driving and intentional/non-intentional non-compliance. While most studies report distracted driving and speeding as primary reasons for crashes [49], the driving behavior in the multi-cultural setting of UAE is unique; about only 18 % of the population is local Emirati while the majority are expatriates, mostly from the Indian sub-continent, south-east Asian countries, and Europe [50]. This is believed to contribute to varying driving behaviors, such as tailgating, swerving, random changing of lanes, and left-side overtaking, among others. For instance, a recent study [14] reported that about quarter of the accidents in Abu Dhabi is caused due to tailgating and other reckless driving behaviors. Also over 50 % of all rear-end crashes in Abu Dhabi is caused because of tailgating [51]. These constitute the most common reckless and non-compliance reasons for crashes in Abu Dhabi.

**Table 3:** Descriptive statistics of continuous variables.

	Injured Age	Road Speed	Manufactured Year	Initiator Age
count	1960	1960	1911	1944
mean	31.494	84.643	2008.096	32.353
std	13.856	27.176	6.033	11.284
min	0	20	1965	4.0
25 %	23	60	2005	24
50 %	29	80	2009	30
75 %	39	100	2013	38
max	115	140	2017	83

With the recent change of women entering the workforce, males remain the majority driving within the emirates [52,53]. Also, most expatriate workers are men who relocated to the UAE for employment. Most individuals prefer to drive with a public transportation system relatively in its infancy, given that both the initiator and the injured gender report reflect a higher percentage of males than females. Also, the most preferred mode of travel in Abu Dhabi is personal vehicles [54]. The hot arid weather reaching up to 53 °C (127 °F) during the summer months discourages the use of other modes of travel such as walking and biking [55] or use of public transit [56]. The most preferred automobile type is SUVs and Sedans, driven by culture and social needs such as large family size and social status. Hence most vehicle types involved in the crashes are SUVs and personal cars.

RoadSafetyUAE [57] reported that young drivers below 30 were responsible for 45 % of all road accidents in the country. In contrast, studies have revealed that novice drivers aged between 18 and 24 contribute to double death rates as compared to older drivers [58]. This study examined only vehicle-to-vehicle crashes. The mean and the median age of the injured and the initiator is around 30 years (Refer to Table 3), while, 75 % of the subjects were under the age of 40. This represents the median age of UAE and the majority of the expatriate workforce population in Abu Dhabi. The designated speed limits in Abu Dhabi and most of the UAE range from 60 kmph to 140 kmph; most internal roads have speed limits between 60 and 80 kmph, while highway speeds typically range from 100 to 140 kmph (See Table 3 and Fig. 6).

### 3.2 Predictive models using ML algorithms

Three main findings were observed in the performance of ML models' when predicting the degree of injury. First, as shown in Table 4, the predictive accuracy of the models ranged from 0.31 (SVM) to 0.44 (RF), which are all higher than a random guess of 0.25 (as there are four categories in the dependent variables). Similar results were observed for the F1 scores, highlighting a good balance in the prediction accuracy of the different classes. The results confirm that the three ML techniques can predict (despite varying accuracy levels) crash severity (the list of variables used to predict crash severity was shown earlier in Table 1). Secondly, the training cross-validation scores are relatively close to the testing scores, showing no signs of overfitting. Therefore, the models still performed well with the test dataset, which was unused during the model training stage. This result indicates good generalizability and flexibility of the algorithms, which are highly desired when applying the models to new datasets. Thirdly, the comparative analysis shows that RF outperformed the other methods in terms of accuracy and F1 scores, particularly SVM. The strong performance of RF (and GB) is mainly attributed to their “ensemble” nature, which builds the prediction based on a collection of classifiers. As seen in Table 4, the total number of classifiers involved in making the final predictions for RF and GB are 600 and 800, respectively.

**Table 4:** ML model prediction performance.

Method	CV Accuracy	CV F1-score	Test Accuracy	Test F1-score	Optimal Hyper-parameter
**RF**	0.3929	0.3876	0.4439	0.4382	Max_depth = 12 n_estimators = 600 min_samples_leaf = 2
**GB**	0.3769	0.3765	0.4031	0.3963	Max_depth = 5 Min_samples_split = 6 N_estimators = 800 Learning_rate = 0.01
**SVM**	0.3272	0.3169	0.3061	0.2883	Penalty = elasticnet Alpha = 0.005 L1_ratio = 0.64 Eta0 = 0.5 Learning rate = adaptive Fit intercept = True

**Fig. 7:**
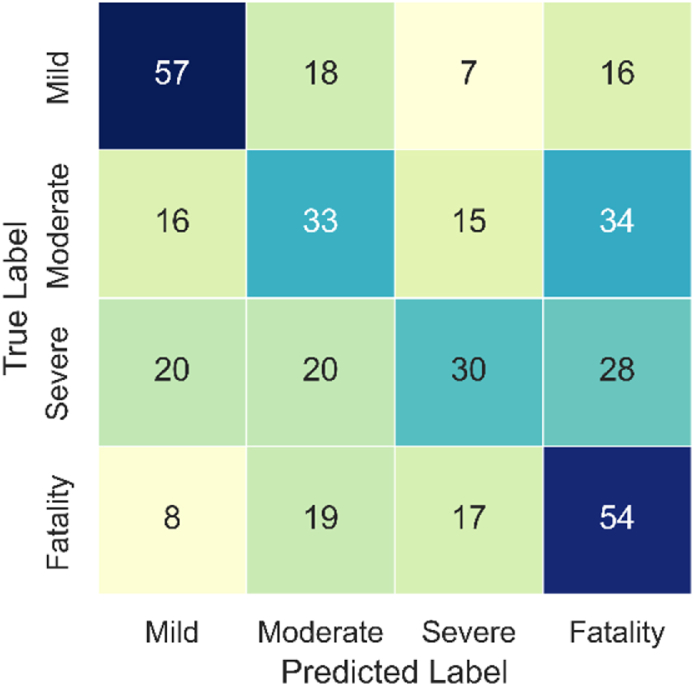
Confusion matrix of the RF model.

**Fig. 8:**
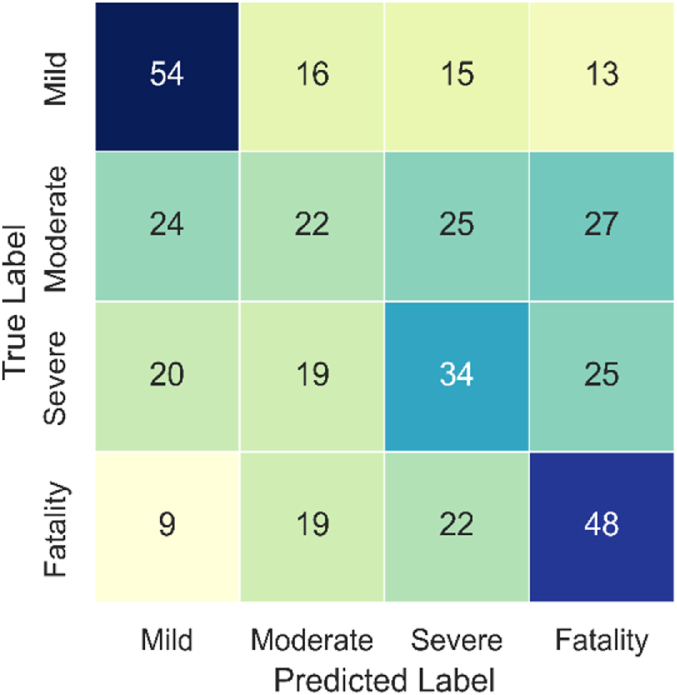
Confusion matrix of the GB model.

**Fig. 9:**
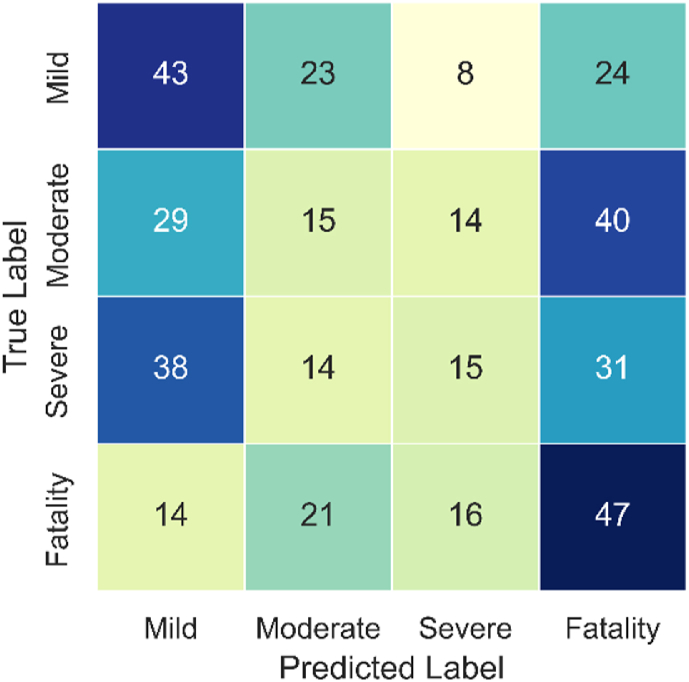
Confusion matrix of the SVM model.

The confusion matrices (Fig. 7, Fig. 8, Fig. 9) confirm the above findings, showing the predicted and actual (observed) values for RD, GB, and SVM models, respectively. The results show that RF and GB have better (i.e., more balanced) distributions when predicting the different classes than SVM. The latter over-predicted the Mild and Fatality classes while under-predicting the other two categories. Here again, the best distribution is seen for the RF model, confirming its higher credibility compared to its counterparts. Given the superior overall performance of RF, it was selected for the feature importance analysis.

**Fig. 10:**
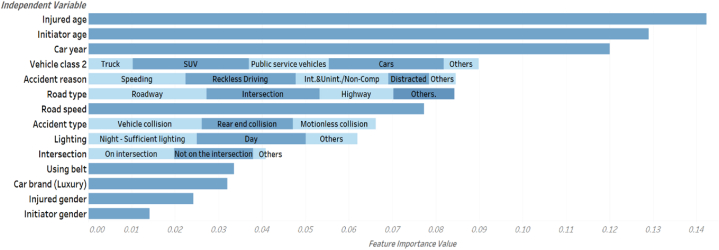
Feature importance values for RF model.

Fig. 10 presents the feature importance analysis conducted on the RF model. A feature importance score is shown for each independent variable based on its contribution to the predicted variable (i.e., crash severity). Results show that the age of the injured and initiator, as well as the manufacturing year of the car show the highest effects on crash severity. To the best of the authors’ knowledge, this is the first study that considers the age of both the injured and initiator of the crash when predicting crash severity. Recent studies using data mining techniques either ignored the age variable [59,60] or did not use the age of the injured as a predictor for the severity of the crash [13],[61].

Conversely, several studies reported the cause of the crash and crash type [48], lighting conditions [61], and gender [12] as significant predictors of the severity of crashes. However, this study reports gender as the least important feature for the crash, crash type, and lighting as slightly associated with the severity of the crash.

#### 3.2.1 Age characteristics

Studies have traditionally used the age of the initiator as a predictor of the severity of crashes, none of these studies used the victim's age to assess the severity of crashes. This study examines vehicle-to-vehicle crashes and the age group of the victims involved. Data from the original dataset reveal that most initiators and injured are between 20 and 30 years old (38.5 % and 33.2 %, respectively). However, the mean and median age of both the initiator and the injured is over 30 years. Henceforth, policies targeting the reduction of traffic crashes should take an age-specific approach to mitigate crash severity. For instance, in the younger age group (<30 years) the frequency of licensing and registration could be increased (from every 10 years to every five years) by each requiring a reassessment of their driving skills, while for the higher age group, the frequency could remain the same with a reassessment of their driving skills.

### 3.3 Vehicular characteristics

While most studies examine the type [11,62], make, and class of the vehicle [14], the age of the vehicle has rarely been inquired for its impact on the severity of crashes. The RF model presents the importance of the age of the vehicle on the severity of crashes, higher than vehicle class or its make. A study by NHTSA [63] reported that cars older than 18 years at the time of the crash are 71% more likely to injure its occupant than cars three years old or less. Conversely, newer vehicles are associated with a reduced risk of crashes and their severity [64]. However, about 89.6 % of the vehicle-to-vehicle crashes in the current study were with cars 18 years or below. This indicates that the multicultural setting of Abu Dhabi and the UAE lends itself to a more complex driving environment. For instance, as stated earlier, with 82 % expat population from the Indian sub-continent, south-east Asia, and Europe nations [50], the country reports an average age of cars in the UAE to be 5.2 years only preceded by Saudi Arabia (3.8 years) and China (5.1 years) [65]. With more expats driving newer and faster cars, with varying driving cultural experiences can lead to unsafe driving environment. A study by Hammoudi et al. [66] reported that in UAE, 65 % of male and 54 % of female drivers confirmed that they had adapted to aggressive driving behaviour and 70 % of male and 71 % female admitted to being sometimes very angry to other drivers on the roads. While the cultural setting remains as is, an approach to deal with this issue is to develop a more stringent driver licensing and training process.

Typical to most cities in the US, UAE reports high vehicle ownership per capita, with about one car per every two residents, higher than London or New York [67]. According to the WHO Road Safety Report [1], about 88 % (2,996,338 of 3,391,125) of the UAE registered vehicles are cars and 4-wheeled light vehicles. The data from this study report that about 79 % of the vehicular crashes recorded in Abu Dhabi are in cars and 4-wheeled light vehicles (typically SUVs), primarily privately owned vehicles. This is also representative of other studies; hence, one of the most recommended mitigation approaches is to enhance the availability and efficacy of public transportation, as well as other alternative modes of travel in the region.

#### 3.3.1 Crash characteristics

The importance of the crash characteristics reports moderate significance in assessing the severity of crashes. The reason for the crash was reported as the highest priority, followed by road type and speed. Studies investigating the severity of crashes in the last two decades have extensively studied the impact of these factors, and the study's outcome aligns with those findings [62,68]. The least important factors in this category were the car brand and the seat belt's reported use. Upon further examination, the car brand was categorized as either standard or luxury with about 60 % classified as luxury. The low significance possibly reflects the limitation of binary classification of the variable. Groping of these vehicles based on their safety ranking may reveal different results. However, contrary to many studies, wearing seatbelt report a low importance on the severity of crashes. The data revealed that no seat belt was observed in 40 % of the crashes in this study. However, only 19 % of those without seatbelt resulted in fatal or severe impact. With over 81 % resulting in moderate to mild impact, the use of seatbelt possibly reported low importance.

#### 3.3.2 Gender

It is well-documented that males are typically high-risk takers compared to women [69,70]. This has been consistently prevalent in traffic crash studies that report a higher probability of men with increased severity of crashes [11]. Although the outcome of this study aligns with other studies, its importance on the severity of the crash is relatively insignificant. While researchers stress the importance of creating gender-specific transportation policies [71], our data indicates that the gender effect in assessing crashes' severity is relatively low.

## 4 Conclusion

The severity of crashes has been a focus of investigation for over two decades, especially with increased vehicle ownership and urbanization of cities and the devastating health and socio-economic consequences worldwide. Despite the above, studies conducted in the UAE context are lacking. This study investigates the severity of road crashes in Abu Dhabi using advanced methodological approaches of ML. Data from the Abu Dhabi Police for 11334 road crashes that occurred between 2014 and 2017 were used for this study.

Advanced methods and approaches of Machine Learning such as Random Forest, Gradient Boosting, and Support Vector Machine are used to investigate the road crashes in Abu Dhabi. After downsampling, Random Forest reported the highest accuracy in predicting the severity of crashes and was used to determine the importance of various factors. The importance evaluation reported that the age of the initiator, the injured, and the vehicle have significant effects on the severity of crashes. Such factors were rarely considered in previous studies in the literature.

This study is the first of its kind to assess the importance of various individual, vehicular, and road characteristics on the severity of crashes in the Emirate of Abu Dhabi using the crash data reported during 2014–2017. Some important implications of this study are.

1. Young adults need to be frequently re-assessed for their driving skills. It is recommended that the duration of driving license for drivers in the age group below 30 should be decreased to 5 years instead of the current 10 years duration. Frequent evaluations should also be mandated, in association with driving violations, to assess driving skills through driving workshops and/or simulated driving tests.
2. Alternative modes of transportation, especially public transit, should be enhanced for their availability and connectivity. UAE is developing a soon-to-be operationalized Etihad Rail Regional passenger train service connecting major cities across different emirates. This is envisioned to reduce the traffic on major highways and hence the possibility of reducing road crashes. An integrated transportation plan has been proposed to enhance the public transportation service in Abu Dhabi, as per the Plan Abu Dhabi 2030 [72].
3. In a multi-cultural setting such as UAE, the role of culture and nationality can add insights to proposing appropriate policies targeting diverse nationalities that are most involved in road crashes. A recent study by Albuquerque and Awadalla [6] reported that 66 % of the crashes are by drivers from Emirates, Pakistan, India and Egypt while drivers from Sweden, America, Australia and Japan account for less than 1 % combined. Also, in the UAE, driving licenses from 33 countries can be presented to directly obtain a UAE driving license, while other nationalities have to pass the written and driving test to obtain a UAE license. This disparity needs to be investigated if such policies impact of road crashes, which warrants further investigation.

While this study brings unique insights into understanding the importance and impact of various factors on the severity of crashes in the UAE, future studies can include emotional factors along with physical and demographic characteristics to better understand the severity of crashes and their implications [73]. The data used for this study was limited to 4 years (2014–2017), which may indicate a change in crash trends since then in the UAE. The limitation of the findings to be relevant for the current trends of crashes is debatable. Recent data can add more insights to the current study. However, while this study is limited to the availability of such data, studies in other regions of the Middle East can build on the current work and investigate the national and expatriate population's role in road crashes [14].

However, it is important to acknowledge the main limitations of the developed ML models as follows: (1) being purely a data driven approach, the model performance is limited to the variation within the variables used; (2) the randomness in sampling the dataset used to train the models; and (3) the selection of independent variables being limited to the available factors within the dataset. As recommended above, other types of factors, such as emotional and physical attributes can impact model performance. Methodologically, it is interesting to note that the continuous variables often exhibited higher importance than categorical and binary variables, which can be attributed to the nature of the “Gini” feature importance metric, which is known to emphasize the rank of high cardinality features, potentially introducing bias in the results [74,75]. Despite this limitation, the results confirm the importance of further analyzing the age of the injured person, the age of the crash initiator, and the age of the car, which are rarely considered in the literature, confirming the novelty of this work. Another important novelty is the coverage of the UAE context, setting the stage for more studies focused on and originating from the GCC region.

## Data availability statement

The data that support the findings of this study are not available due to privacy restrictions on data sharing by the original entity [AD Police] and adhering to privacy restrictions of the indicents of accidents collected in Abu Dhabi. Henceforth, the authors do not have permission to share data of this study.

## CRediT authorship contribution statement

**Praveen Maghelal:** Conceptualization, Investigation, Methodology, Resources, Supervision, Writing – original draftWriting – original draft, Writing – review & editingWriting – review & editing. **Abdulrahim Haroun Ali:** Formal analysis, Software, Validation, Visualization. **Elie Azar:** Formal analysis, Investigation, Software, Supervision. **Raja Jayaraman:** Supervision, Validation. **Kinda Khalaf:** Data curation, Resources.

## Declaration of competing interest

The authors declare that they have no known competing financial interests or personal relationships that could have appeared to influence the work reported in this paper.
